# Design of an extensive information representation scheme for clinical narratives

**DOI:** 10.1186/s13326-017-0135-z

**Published:** 2017-09-11

**Authors:** Louise Deléger, Leonardo Campillos, Anne-Laure Ligozat, Aurélie Névéol

**Affiliations:** 1 French National Institute for Agricultural Research (INRA), Domaine de Vilvert, Jouy en Josas, Paris, 78352 France; 2 LIMSI, CNRS, Université Paris - Saclay, Rue John von Neumann, Orsay, 91405 France; 3 ENSIIE, 1 square de la résistance, Évry Cedex, 91025 France

**Keywords:** Knowledge representation, Clinical natural language processing

## Abstract

**Background:**

Knowledge representation frameworks are essential to the understanding of complex biomedical processes, and to the analysis of biomedical texts that describe them. Combined with natural language processing (NLP), they have the potential to contribute to retrospective studies by unlocking important phenotyping information contained in the narrative content of electronic health records (EHRs). This work aims to develop an extensive information representation scheme for clinical information contained in EHR narratives, and to support secondary use of EHR narrative data to answer clinical questions.

**Methods:**

We review recent work that proposed information representation schemes and applied them to the analysis of clinical narratives. We then propose a unifying scheme that supports the extraction of information to address a large variety of clinical questions.

**Results:**

We devised a new information representation scheme for clinical narratives that comprises 13 entities, 11 attributes and 37 relations. The associated annotation guidelines can be used to consistently apply the scheme to clinical narratives and are https://cabernet.limsi.fr/annotation_guide_for_the_merlot_french_clinical_corpus-Sept2016.pdf.

**Conclusion:**

The information scheme includes many elements of the major schemes described in the clinical natural language processing literature, as well as a uniquely detailed set of relations.

## Introduction

The progressive adoption of electronic health records (EHRs) is paving the way towards making available large amounts of data for research. Raw EHR data may be transformed into clinically relevant information and then be used in traditional or translational research [1]. Natural language processing is essential to phenotyping EHR data because of the amount of clinical information buried in the narrative content.

The path towards EHRs is nonetheless not free of challenges [1]. One of the hurdles is the coexistence of several information models for representing clinical information available in EHRs. The Clinical Document Architecture (CDA) in the Health Level 7 (HL7) framework coexists with the Clinical Element Model (CEM) [2] and other standards such as the openEHR [3] and the ISO 13606 [4].

Developing equivalent clinical models is a key element to achieve the semantic interoperability of EHR systems [5]. The Clinical Information Modeling Initiative (CIMI) [6] and the SemanticHealthNet (SHN) [7] initiative are international efforts towards this goal. It can be argued that information models rely on terminologies that specify the concepts used in the model [8]; for instance, medications in RxNorm10 or clinical terms in SNOMED CT. However, information and terminology models tend to be designed by different groups with dissimilar data structures. Some researchers have indeed attempted to validate the use of terminologies in EHR-based standards (e.g. SNOMED CT in the HL7 Clinical Document Architecture) [9].

In this paper we will focus on a text-based representation of information that is text-anchored (i.e. mentions of clinical entities) or that may be derived from text data (e.g. relations between entities identified in clinical texts). Addressing the unification of information models is out of our scope here. Our goal is to put forth a representation scheme that will support secondary use of EHR data for conducting a large variety of retrospective studies. More specifically, we aim to support information extraction from clinical narratives in order to answer clinical questions such as: “What is the prevalence of incidental findings in patients with suspected thromboembolic disease?”, “What is the contribution of CT venography in the diagnosis of thromboembolic disease?” or “What are the types and grades of toxicities experienced by colon cancer patients receiving FOLFOX therapy?”.

Simple and fast low-level annotations have already yielded good results in mining large datasets, as exemplified in LePendu et al.’s study on myocardial infarction adverse drug effects in rheumatoid arthritis [10]. Another study showed the benefit of exploiting medical concepts, modality and relations between concepts extracted from clinical narratives for accurate patient phenotyping [11]. Furthermore, recent research has shown that information extraction from unstructured clinical narratives is essential to many clinical applications, including secondary use of EHRs for clinical trial eligibility [12].

Overall, the information representation landscape broadly includes two types of representations. First, ontologies or encyclopedic representations that are very detailed and removed from any direct application, with the goal of providing a formal representation of domain or subdomain knowledge. Second, a number of text-based representations of information that are very-well suited to an application they were designed for. Our need is for a representation scheme with a broad scope that remains close to applications grounded in clinical text. The goal is to identify a representation that may connect easily with major knowledge sources used in clinical Natural Language Processing, while covering many aspects of clinical knowledge covered in EHR narratives. We conducted a review of annotation projects and associated annotation schemes for clinical narratives. We found that while all existing schemes had merit, no single scheme covered all the aspects of knowledge representation that we sought, in particular with respect to fine-grained relations between clinical concepts. We then designed a new information representation scheme that related to existing schemes and attempted to integrate best representation practices.

This article describes a new information representation scheme devised from on-going analysis of clinical narratives. This scheme has been applied to annotate a large corpus of French clinical reports described in [13], but is intended to be generally applicable to clinical narratives in several languages and medical specialties. The contribution of this paper is two-fold: first, we present an extensive review of annotation projects and associated annotation schemes for clinical narratives. Second, we provide material for the annotation of clinical narratives, including a new annotation scheme, companion annotation guidelines, and insight on how to devise an annotation methodology for a new project.

## Background

### Representation of information in clinical text corpora

Ethical issues need to be considered before carrying out research on clinical narratives. Privacy issues require supplementary measures to de-identify patient data before releasing the corpus for research. De-identification is usually performed by removing or replacing Personal Health Identifiers with surrogates [14]. This is one of the reasons why clinical corpora are less available than corpora in the biological domain [15, 16].

Improvements in clinical information processing have been reported by adopting adequate annotation frameworks [11, 15, 17–19]. These have been developed in two levels of representation. A low-level annotation is concerned with linguistically and clinically grounded representations to use within a document. This level is concerned with defining (in annotation guidelines) mentions of clinical and linguistic interest, and then marking these instances in clinical text. Most annotation efforts in the biomedical NLP community have followed this trend, especially within the organisation of research challenges.

The second level of representation is a high-level annotation that prioritizes formally integrating all the annotated linguistic and clinical data. That is, this level prioritizes processing the annotated information for reasoning over the whole EHR in a computationally actionable way. Within the context of the Strategic Health IT Advanced Research Project (SHARPn), [20] and [21] developed a higher-level formal (OWL) clinical EHR representation (implemented in cTAKES [22]). This representation is based on the low-level annotation framework explained in [23]. The SHARPn normalized data has been thus converted automatically to the Resource Description Framework (RDF) format by using the CEM-OWL specification. The Biological Expression Language (BEL) [24] seems to be a mix between the low and high-level of annotation for life science text (vs. clinical).

Our work has carried out a low-level annotation, but our scheme can likely be compatible with a high-level representation in the long-run. In the following section, we will review other low-level annotation frameworks of clinical corpora.

### Related work

In this section, we focus on well-documented frameworks issued from medium-scale projects, or schemes that have been widely used in shared tasks or challenges. Additional examples of annotation efforts of clinical data for specific applications or experiments, where the representation scheme or annotation work is not the main focus, are reported in [25–36] (inter alia). These will not be reviewed in detail herein, as we chose to provide an in-depth analysis of efforts providing rich annotation guidelines that we relied on to build our own scheme. We refer the readers to a recent review of the litterature in clinical NLP for a more complete overview of the field [37].

We review the annotation schemes outlined in Table 1, in chronological order. Note that we classified the annotations in the Informatics for Integrating Biology and the Bedside (i2b2) challenges as entities or attributes, in order to make clearer the comparison between schemes. However, this distinction does not exist in some i2b2 references [38–42].

**Table 1:** Annotated clinical corpora and/or annotation guidelines (*tks* stands for *tokens*)

Reference	Text type/size	Entities/events	Mapping	Attributes	Relations	Temporal data
Ogren et al. [17]	160 clinical notes (47,975 words)	1 (*disorder*)	SNOMED	3 (*context, status, flag*)		
CLEF [15]	Cancer patient records (50 clinical narratives, 50 histopathology and 50 imaging reports)	6 (*condition, locus, intervention, investigation, result, drug*, *device*)	UMLS	3 (*negation, laterality, sub-location*)	5 (*has target, has finding, has indication, has location*, *modifies*)	Derived from TimeML
i2b2 [38–42, 46]	2009 data: 1,243 discharge summaries 2010 data: 1,748 discharge summaries and progress reports 2012 data: 310 discharge summaries (178,000 tks)	7 in the 2009 call (*drug name, dosage, mode, frequency, duration, reason*, *list/narrative*) 3 in the 2011 call (*tests, treatments, problems*) 6 in the 2012 call (*tests, treatments, problems, clinical department, evidential, occurrence*)		6 values for assertion in the 2011 call (*present, absent, possible, conditional, hypothetical* and *not associated with the patient*). In the 2012 call, attributes were *type*, *polarity* (*positive, negative*) and *modality* (*present, proposed, conditional, possible*)	8 in the 2011 call (*improves, worsens, causes, is administered for, is not administered because of, reveals, conducted*, *indicates*)	Derived from TimeML
IxA-Med-GS [19]	75 clinical reports (41,633 tks)	3 (*disorder, drug, procedure*)		2 (*negation, speculation*)	2 (*caused by, related with*)	
THYME [47–49]	1,251 clinical notes (colon and brain cancer)	All events related to the patient’s clinical timeline (e.g. procedures, diseases, diagnoses or patient complaints)		7 (*docTimeRel, type, polarity, degree, contextual modality, contextual aspect*, *permanence*)	5 types of temporal links (*TLINKs*) and 4 types of aspectual links (*ALINKs*)	Derived from TimeML
SHARP Annotation Templates [23]		7 (*diseases*, *signs*/ *symptoms, procedures and methods, anatomical sites, medications, devices*, *labs*)	UMLS (SNOMED and RxNorm)	General (13), lab (7), medication (13) and relation attributes (3)	13 (*affects, causes, complicates, contraindicates, degree of, diagnoses, disrupts, is indicated for, location of, treats, manifestation of, prevents*, *result of*)	THYME guidelines
MiPACQ clinical corpus [18]	Clinical narrative and pathology notes (colon cancer, 127,606 tks)	17 (15 UMLS groups + *signs*/ *symptoms* + *person*	UMLS	2 (*negation, status*)		
ShARe/CLEF eHealth labs [51, 52]	433 clinical reports (discharge summaries, radiology, electrocardiograms, echocardiograms)	1 (*disorder*)	UMLS	10 (*negation, uncertainty, subject, course, severity, conditional, generic, body location, docTime*, *temporal*)		
Harvey [53]	750 primary care notes (22,914 tks)	4 (*quantity, locative, temporal*, *on examination*)				

The Mayo Clinic group prepared a gold standard corpus of 160 clinical notes (47,975 words) [17]. Entity types were restricted to Disorders—Signs or Symptoms were excluded—and mapped to the Systematize Nomenclature of Medical Terms (SNOMED-CT). Three types of disease attributes were labelled: context (*current, history of*, and *family history of*), status (*confirmed, possible* and *negated*) and a flag for conditions unrelated to the patient. The task involved four annotators in a pair-wise annotation workflow with consecutive rounds to achieve consensus annotations.

The Clinical E-Science Framework (CLEF) corpus [15] gathered anonymized cancer patient records (50 clinical narratives, 50 histopathology reports and 50 imaging reports). Around 25 annotators participated. Files in XML format included annotations of six types of entities (*condition, intervention, investigation, result, drug or device* and *locus*) and three attributes (*negation*, for *condition*; *laterality*, for *locus* or *intervention*; and *sub-location*, for *locus*). The entity types were mapped to the Unified Medical Language System ^®;^ (UMLS ^®;^, [43]) semantic types. Relations between entities spanned over different sentences and involved five types: *has target, has finding, has indication, has location*, and *modifies* (to link attributes with entities). Temporal relations were also encoded between temporally located CLEF entities (TLCs) and time expressions (dates, times and durations), which were normalized following the TimeML TIMEX3 standard [44]. Time relations were marked as CTlink annotations with the following types: *before, after, overlap, includes, ended-by, begun-by, is-included* and *unknown*.

The Informatics for Integrating Biology and the Bedside (i2b2) challenges provided the community with richly annotated corpora of de-identified clinical texts (discharge summaries and progress reports). The i2b2 2009 task focused on extracting medication data (*drug name, dosages, modes, frequencies, durations, reasons* and *list/narrative*) [38]. The corpus contained 1243 discharge summaries [45]. In the i2b2 2010 competition [39], *problems, treatments* and *tests* were annotated (see more details in the guidelines, [40–42]). The 2010 data were 394 training reports, 477 test reports, and 877 unannotated reports. The 2012 call [46] widened the encoding of the aforementioned events to *clinical department, evidential* and *occurrence*. The *polarity* (*positive* or *negative*) and the *modality* of events (*factual, conditional, possible* or *proposed*) were also considered. For problem annotations, *assertions* were marked and classified (*present, absent, possible, conditional, hypothetical* and *not associated with the patient*). Relations were encoded within the same sentence, between a treatment (T) and a problem (P) (T *improves* P, T *worsens* P, T *causes* P, T *is administered for* P, or T *is not administered because of* P), between a test and a problem (test *reveals* P or test *is conducted for* P), and between two problems (P *indicates* P). Pronominal and lexical coreference was annotated with relation to entities and persons in the form of coreference pairs (concepts or pronouns from the same class). The i2b2 2012 competition involved the annotation of time expressions (in the TIMEX3 format) and marking of their type (date, time, duration and frequency), normalized value and modifier. Temporal relations (or temporal links, *TLINKs*) of seven types were annotated and finally merged into three (*before*, *overlap* and *after*). Two types of section times were labelled (*admission* and *discharge*). The 2012 data consisted of 310 discharge summaries (178,000 tokens).

The THYME (Temporal Histories of Your Medical Events) project [47] is an ongoing effort to annotate temporal information in clinical data. The data reported in [48] consists of 1251 de-identified clinical notes on colon and brain cancer. The annotation guidelines [49] do not define specific entity types. Any event or state relevant to the patient’s clinical timeline was annotated (procedures, diseases, diagnoses, patient complaints or states). The annotation scheme regarding event attributes is rich. The *DocTimeRel* attribute encodes the temporal relation between the event in question and the time when the record was authored (*before, after, overlap* or *before/overlap*). The *type* attribute may bear a *default* value or may be *aspectual* or *evidential*. There are other attributes marking *polarity*, *degree*, *contextual modality*, *contextual aspect*, and *permanence*. Time expressions were normalized following the TIMEX3 formalism and classified into six types: *date, time, duration, quantifier, prepostexp* (e.g.*preoperative*) and *set* (which is related to frequency). Temporal links (*TLINKs*) were annotated between two events or an event and a TIMEX3, applying the following values: *before, contains, overlap, begins-on* and *ends-on*. Finally, aspectual links (*ALINKs*) were tagged between an aspectual and a non-aspectual event in the same sentence. ALINK labels were *continues, initiates, reinitiates* or *terminates*.

The SHARP Template Annotations [23] aim at normalizing relevant clinical mentions to the Clinical Element Model (CEM). Entities are mapped to UMLS Concept Unique Identifiers (hereafter, CUIs) through RxNORM (for drugs) and SNOMED (for everything else). Entities are restricted to a 7-category typology (*diseases, signs or symptoms, procedures or methods, anatomical sites, medications, devices* and *labs*) and their attributes are richly specified. There are 13 general attributes (e.g. *body side, course* or *severity*) together with seven lab-related attributes (e.g. *value number* or *lab interpretation*) and seven medication attributes (e.g. *dosage, strength* or *route*). Relations are limited to entities inside sentence boundaries, and 13 types are contemplated: *affects, causes, complicates, disrupts, contraindicates, degree of, diagnoses, is indicated for, location of, manages/treats, manifestation of, prevents* and *result of*. Relation attributes are also tagged (*conditional, negation* and *uncertainty*). Pronominal and lexical coreference is encoded according to two types (*identity* and *apposition*). Lastly, the annotation of temporal information is compatible with the THYME guidelines.

There exists a de-identified corpus of 127,606 tokens of clinical narrative and pathology notes on colon cancer. It was gathered for developing the MiPACQ (Multi-source Integrated Platform for Answering Clinical Questions) question answering system [18]. A distinctive feature of the MiPACQ corpus is its layered annotations, featuring treebanking, predicate-argument structure (PropBank) and semantic entity labelling. Semantic annotation of entities followed the UMLS typology of semantic groups [50], thereby avoiding the ambiguity between semantic types. An exception was the SignOrSymptom type, which was distinguished from Disorders. The Person category was also added to the scheme. Entity attributes comprised two slots: *negation* (*true* or *false*) and *status* (*possible, history of, family history of*, or *none*).

The ShARe/CLEF eHealth evaluation labs (2013-2014) [51, 52] fostered the annotation of disease mentions in 433 clinical reports (discharge summaries, radiology, electrocardiograms and echocardiograms). Entities in the Shared Annotated Resources (ShARe) scheme belonged to all semantic types in the disorder semantic group (except Findings) and had to be mapped to UMLS CUIs. The typology of attributes to provide was heterogeneous: *negation* and *uncertainty indicators*, *subject* (i.e. the entity is related to the patient in question), *course* (i.e. the alteration or evolution of the condition), *severity, conditional, generic* (i.e. a non-specific mention of a disease), *body location, docTime class* (i.e. the time relation between a disease and the moment when the report was written) and *temporal expression* defined according to the TIMEX3 TimeML standard (*start date, duration* and *end date*).

The more recent IxA-Med-GS corpus [19] contains 75 clinical reports in Spanish (41,633 tokens) annotated with adverse drug reactions (ADR). *Disorder* (including symptoms), *drug* and *procedure* entities were labelled, with attributes for *negation* or *speculation*. ADR events are indicated as *caused by* and *related with* relations.

Finally, the Harvey corpus comprises 750 de-identified primary care notes (around 17,656 words, 22,914 tokens), which have been annotated with both syntactic chunks and semantic information [53]. Syntactic phrases bear Part-of-Speech (PoS, hereafter) tags. Semantic annotation is performed on four types of entities: *quantity expressions, temporal expressions, locative expressions* and *on examination expressions*. For further references on PoS tagging of clinical records, we refer to [53, 54].

### Comparison of annotation frameworks

As pointed out by Wu et al. in a study of negation accross four corpora [55], there are several levels of differences in annotations across guidelines: a level related to the classes of entities annotated (i.e. the semantic types considered), a level regarding the span of the annotation, and a level of presence/absence of overlapped annotations.

With regard to the elements annotated, these are generally entities or events, relations between them, and their attributes or values. Depending on the granularity of the annotation—according to the task for which the corpus was devised—entities/events are mapped to the Unified Medical Language System, and assertion, aspectual, temporal and coreference information are also encoded. However, the element types (i.e. entities, attributes or relations) used to encode the same information are not always similar across schemes. For example, the entity *locus* in the CLEF scheme approximately corresponds to the attribute *Body_Location* in the ShARe framework. Few corpora include tree-banking and predicate-argument structure (PropBank) (e.g. [18]), or annotation of syntactic chunks and Part-of-Speech (PoS, hereafter) (e.g. [53, 54]).

The rules for defining the type of text mentions marked as entity annotations can differ between frameworks. For instance, only noun phrases are annotated in MiPACQ. In contrast, the guidelines for the i2b2 challenge consider noun and adjective phrases (including articles or possessives). The annotation span can exclude function words in specific frameworks; e.g. MiPACQ does not include possessive adjectives. As a result, the phrase *her chest x-ray* would be annotated completely as a Test according to the i2b2 guidelines, while only the portion *chest x-ray* would be annotated as a Procedure according to MiPACQ guidelines.

The THYME scheme rules out prepositions in temporal annotations (prepositions are regarded as *signals* of temporal events; however, annotations encompass other phrase categories). Other schemes are broader. The SHARP initiative considers the longest string matching UMLS terms—including disjoint annotations (e.g. *his face was weak* ≈*facial paresis*, C0427055). This is similar to the ShARe Template annotations, which focus on the more specific terms (e.g. *lung cancer* instead of *cancer*). Lastly, the Harvey corpus annotated syntactic chunks corresponding to noun, adjective, adverbial and verb phrases.

As for embedded annotations, the SHARP framework includes subspans of annotations belonging to different semantic types. The Harvey corpus considers embedded annotations only when a phrase chunk or a main verb is included in an expression (or vice versa) and excludes partial overlapping.

Finally, we can also compare annotation frameworks with regard to whether they take into account linguistic knowledge, domain knowledge (or expert annotation [30]), or both. Most initiatives do not rely on domain knowledge, because annotators are not health professionals. For example, the SHARP or ShARe schemes rule out inferred relations (i.e. based on medical reasoning) unless authorized. There are nonetheless some exceptions. Both clinicians and non-clinicians annotated the CLEF and IxA-Med-GS corpora, whereas only medical students annotated the i2b2 resources and the Harvey corpus.

### Caveats and lessons learned from existing frameworks

The review of prior work on existing framework and their use for annotating clinical narratives highlights specific features of frameworks and their applications that must be taken into account for the use of existing frameworks and annotated datasets, or the design of new material:

- *Category definition.* The definition of some concept categories varies between frameworks. One prominent category that exhibits definition variation is a category refered to as Disorders or Medical problems. One source of One source of definition variation is whether any medical condition experienced by patients should be included in a broad category of whether diagnosed Diseases should be separated from the clinical Signs or Symptoms leading to the diagnosis.
- *Entity span.* When translating a framework representation to text instantiations of the framework elements, different levels of strickness are observed in the marking of entities. Markable entities are sometimes restricted by linguistic categories, may cover continuous or discontinuous text spans, may overlap with other entity types from the framework, and so on.
- *Attribute anchoring.* When attributes associated to entities are marked, there may be a requirement to also mark a text clue or segment supporting the assigment of the attribute.

Overall, it seems that the more information can be encoded, the better information representation can be obtained, and the better the interoperability with other schemes. However, this may come at the expense of the time spent designing a framework and anticipating a variety of use cases for it, as well as the time spent to carry out an annotation campaign.

## Methods

The scheme built on prior work as much as possible while trying to avoid some of the caveats reported and adapt to the nature of our data.

One major source of biomedical knowledge we relied on is the UMLS. We specifically used the Semantic Network, which consists of a set of broad categories (or Semantic Types) that provide a consistent categorization of all concepts in the UMLS Metathesaurus, and a set of relationships (or semantic relations) that exist between semantic types. We also used the UMLS Semantic Groups, which are a coarser-grained set of Semantic Type groupings designed according to principles of semantic validity, parsimony, completeness, exclusivity, naturalness, and utility [50, 56]. The annotation scheme for entities relied on the UMLS Semantic Groups (hereafter, SGs) while relations were derived in part from the UMLS Semantic Network. The scheme for temporal annotation was derived from the TimeML standard [44] as well as previous temporal annotation in clinical data, such as i2b2 [39] and THYME [48].

The final scheme was intended to be suitable for many clinical subfields. In preliminary work, we tested its applicability to clinical notes covering a range of specialities, including foetopathology [57]. The annotation scheme was designed to provide a broad coverage of the clinical domain, in order to annotate medical events of interest mentioned in the clinical documents.

## Results

**Fig. 1:**
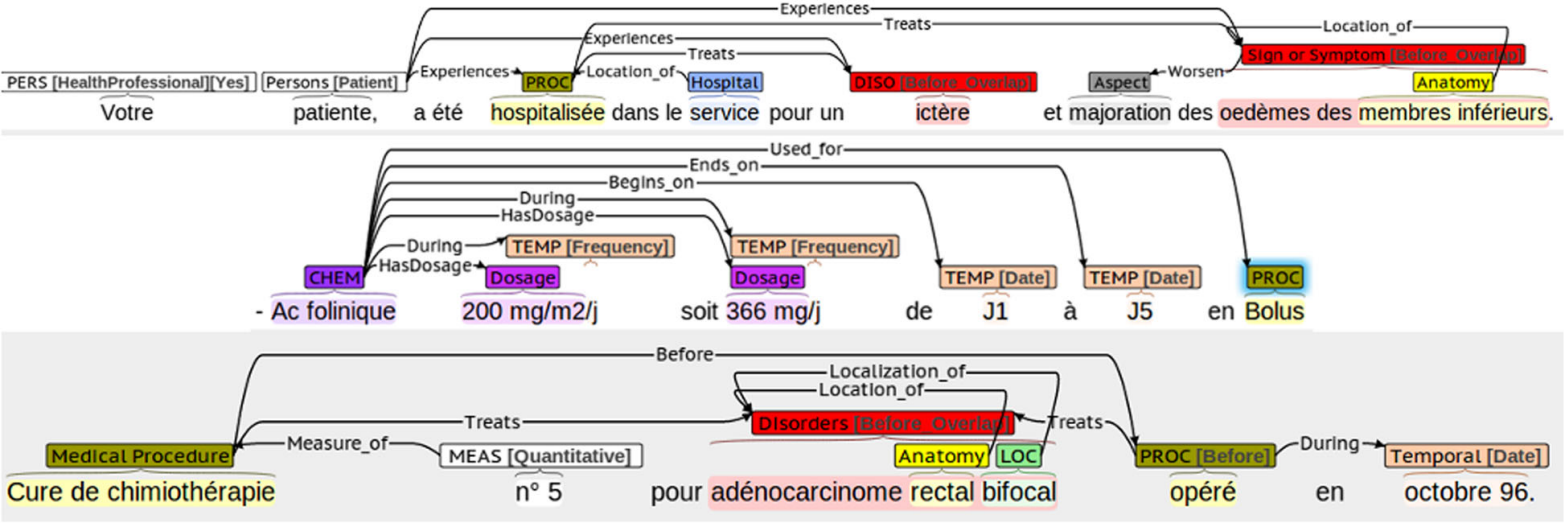
Sample annotations of clinical narratives using the scheme

In this section, we describe the elements of the annotation scheme. Semantic annotations in the scheme include entities, attributes, relations between entities, and temporal annotations. Figure 1 shows some examples of annotated clinical text.

### Entities

The annotation scheme for entities comprises 13 elements (Table 2). Our scheme was derived in part from the UMLS Semantic Groups introduced by Bodenreider and McCray [50, 56]. We included 9 of the 15 UMLS Semantic Groups: Anatomy, Chemicals and Drugs, Concepts and Ideas, Devices, Disorders, Genes and Molecular Sequences, Living Beings, Physiology and Procedures. Note that the semantic type (hereafter, STY) Findings was not included in the Disorder class, because prior work has shown that it yields many false positives [58, 59]. We also created four additional categories for annotating elements of clinical interest:

**Table 2:** Entities

Entity type	Definition	UMLS semantic type(s)	Examples
Anatomy	Any part or component of the body	Anatomical Structure, Body Location or Region, Body Part Organ or Organ Component, Body Space or Junction, Body Substance, Body System, Cell, Cell Component, Embryonic Structure, Fully Formed Anatomical Structure, Tissue	*foot; right femoral artery*
Biological- ProcessOrFunction	A process or state occurring naturally or as a result of an activity	Biologic Function; Cell Function; Genetic Function; Molecular Function; Natural Phenomenon or Process; Organ or Tissue Function; Organism Function; Physiologic Function	*transit*
Chemicals_ Drugs	Matter of particular or definite chemical constitution; a substance used as a medication or in the preparation of medication	Antibiotic; Biomedical or Dental Material; Carbohydrates; Chemical; Chemical Viewed Functionally; Chemical Viewed Structurally; Clinical Drug; Hazardous or Poisonous Substance; Inorganic Chemical; Pharmacological Substance; Vitamin	*insulin; steroids; Percocet*
Concept_Idea	An abstract or generic idea generalized from particular instances	Classification, Conceptual Entity, Functional Concept, Group Attribute, Idea or Concept, Intellectual Product, Language, Qualitative Concept, Quantitative Concept, Regulation or Law, Spatial Concept	*weight; length*
Devices	An object for diagnosis or treatment	Devices	*pacemaker*
Disorder	A condition of the patient that impairs normal functioning and is manifested by distinguishing signs and symptoms	Acquired Abnormality; Anatomical Abnormality; Cell or Molecular Dysfunction; Congenital Abnormality; Disease or Syndrome; Experimental Model of Disease; Injury or Poisoning; Mental or Behavioural Dysfunction; Pathologic Function; Neoplastic Process	*diabetes; myocardial infarction*
Genes/Proteins	A gene is defined as the portion of DNA encoding the blueprint for constructing a protein	Amino Acid, Peptide or Protein; Enzyme, Lipid; Immunologic Factor; Indicator, Reagent, or Diagnostic Aid; Gene or Genome; Nucleic Acid, Nucleoside or Nucleotide; Receptor	*PTX1; fibrin*
Hospital	Health care facility, office or ward	_	*Mercy Hospital; ER*
LivingBeings	An individual form of life that is not human	Alga; Amphibian; Animal; Archeon; Bacterium; Bird; Fish; Fungus; Invertebrate; Mammal; Organism; Plant; Reptile; Rickettsia or Chlamydia;Vertebrate; Virus	*salmonella, HIV*
MedicalProce- dure	An activity relating to the practice of medicine or the care of patients	Diagnostic Procedures; Health Care Activity; Laboratory Procedure; Therapeutic or Preventive Procedure	*angiography, psychiatric consult*
Persons	Human living beings	Human	*patient; Dr Smith*
Sign/Symptom	A manifestation of a condition	Sign or Symptom	*pain; cough*
Temporal	Temporal expressions	Temporal Concept	*weekly, 1984*

- SignOrSymptom: according to prior work [18, 23], we split Signs and Symptoms and Disorders in separate categories.
- Persons: we created a separate category for human entities and excluded them from the Living Beings group (a choice also made in MiPACQ [18])
- Hospital: we added an item for healthcare institutions [39]
- Temporal: we created a separate category for temporal expressions and excluded them from the Concept and Ideas group, in favor of specific representation of time [44].

We considered a subset of entities as events, namely: Disorder, SignOrSymptom, MedicalProcedure, Chemical_Drugs, BiologicalProcessOrFunction and Concept_Idea. *Event entities* are annotated with specific attributes (e.g. DocTime) and participate in temporal relations (with another event or a temporal expression).

We have not restricted the annotation to UMLS entities or specific syntactic classes (e.g. noun or adjective phrases). When required, we have annotated verbs (e.g. *saigner*, ‘to bleed’), mapping them semantically to UMLS concepts.

**Fig. 2:**
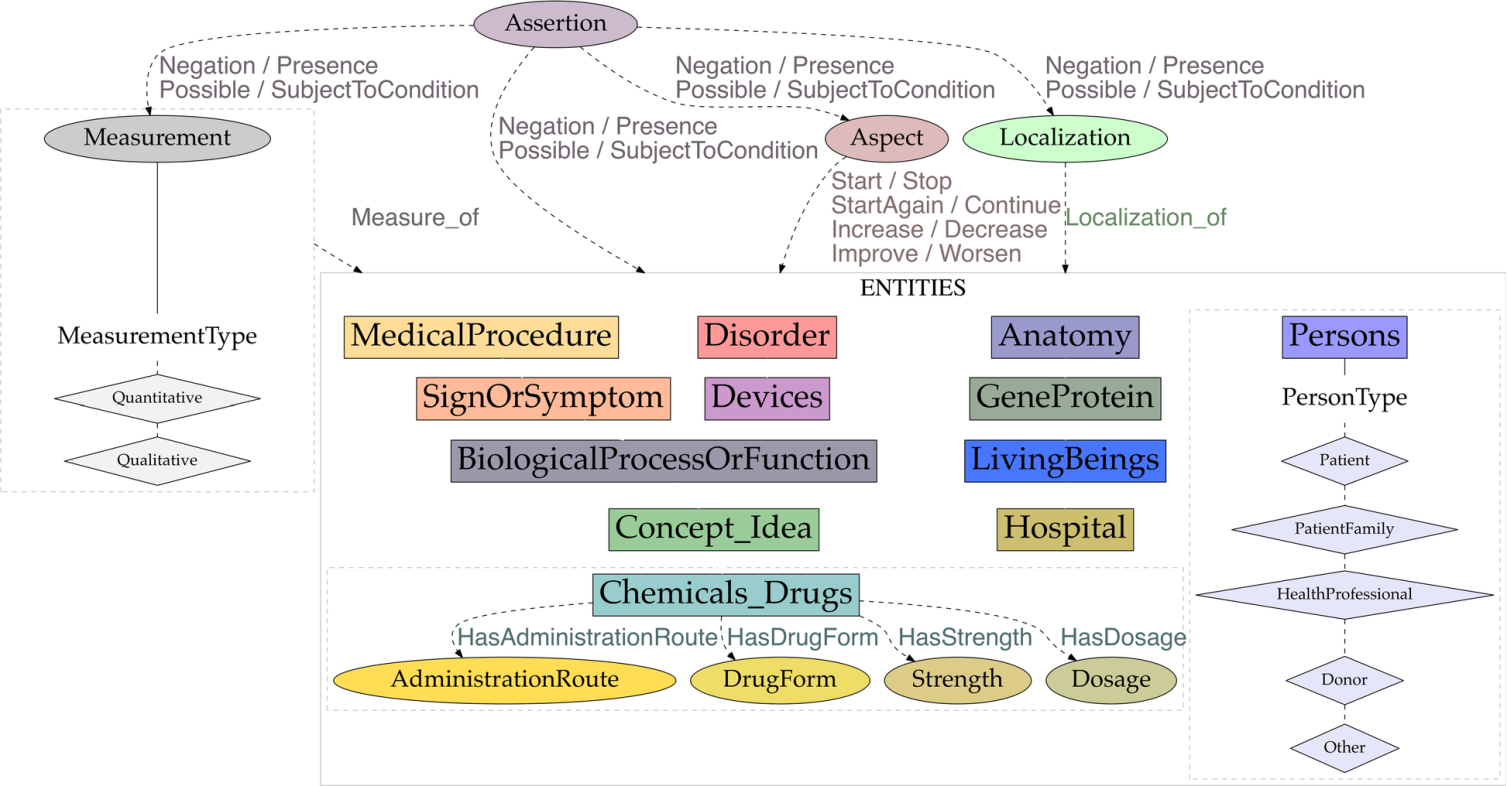
Entities and their attributes

The annotation scheme also defines some attributes (Table 3), which are linked to entities and/or other attributes. Figure 2 shows entities and their attributes. Attributes may have a textual anchor (represented as ellipses) or may consist of a normalized value from a predefined list (represented as diamonds).

**Table 3:** Attributes

Attribute type	Definition	Involved entities	Involved relation(s)	Examples
Aspect	A phrase that represents a change or an evolution (movements of object are not covered)	All entities	Start Stop StartAgain Continue Increase Decrease Improve Worsen	*started on;* *interrupted;* *relapse;* *continued;* *increase in;* *decreased*
Assertion	A phrase indicating a statement of fact or possibility regarding an entity	All entities and Aspect, Measurement and Localization	Negation Presence Possible SubjectTo- Condition	*no;* *presence of;* *suspected;* *in case of*
Localization	Precise area where an entity is located (e.g. body side)	All entities	Localiza- tion_of	*left; bilateral*
Measurement	A figure, extent, attribute or amount obtained by measuring or observing, including subjective qualifications. Two subtypes: Quantitative and Qualitative	All entities	Measure_of	*3 cm; normal*
PersonType	Person entity type; the predefined options are Patient, PatientFamily, HealthProfessional, Donor and Other	Persons	Experiences	*Dr. Colin*
DocTime	Temporal data of an annotated event with regard to the moment when the document was authored; the predefined options are Before, After, Overlap and Before_Overlap	Events		*opération de 1984* [Before]
TemporalType	Type of temporal expression; the predefined options are Date, Time, Duration and Frequency	Temporal	Temporal relations	*1981, deux fois par jour*
Medication attributes				
Administration Route	Route or method of administering a medication	Chemicals/ Drugs	HasAdminis-trationRoute	*oral; IV*
Dosage	How many of each drug the patient is taking	Chemicals/ Drugs	HasDosage	*3 tablets; two puffs*
DrugForm	Form of a medication	Chemicals/ Drugs	HasDrugForm	*tablet; cream*
Strength	Strength number and unit of a prescribed drug	Chemicals/ Drugs	HasStrength	*10 mg; 5 mg/ml*

### Attributes

Wherever possible, we have chosen to enable the marking of text segments that support the assigment of attributes. As a result, some attributes are represented in our scheme as having both attribute and relation elements.

We have flagged abbreviations and acronyms as entity attributes for all entity types. For instance, the mention *HTA* standing for *hypertension artérielle* (‘hypertension’) can be represented as a disorder entity with the attribute “abbreviation”.

**Fig. 3:**
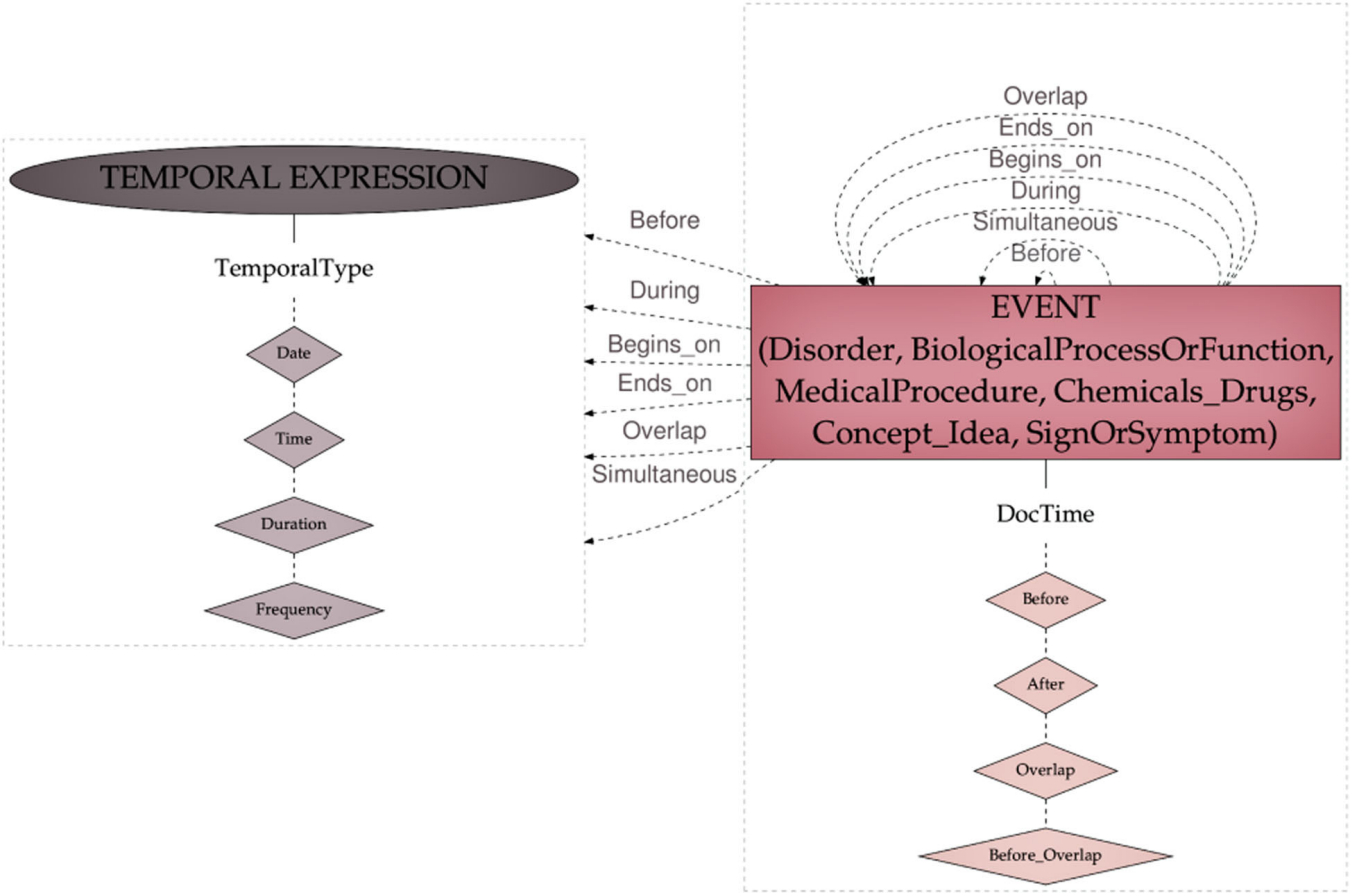
Temporal scheme

Some attributes can be related to any event entity, either directly (DocTime) or using specific relations (Aspects, Assertion):

- Aspect: They are anchors of aspect relations to entities (described below). Aspects are markers that indicate the presence of an aspectual relation. For instance, in the phrase *introduction de l’EPO le 12/04/2012* (‘EPO was introduced on 04/12/2012’) the mention *introduction* (‘introduced’) can be represented as an aspectual marker indicating the Start of the Chemicals_Drugs event *EPO*, meaning that the patient started this drug therapy.
- Assertion: Textual anchors of assertion relations to entities (described below). For instance, in the phrase *pas de douleur* (‘no pain’), the mention *pas de* (‘no’) can be represented as a textual marker indicating the negation of the SignOrSymptom concept *douleur* (‘pain’).
- DocTime: temporal data of events with regard to the moment when the text was created: After, Before, Before_Overlap and Overlap (Fig. 3).

Another subset of attributes are specific to some entities:

- Medication attributes: we simplified the medication attributes in SHARP and defined four types: AdministrationRoute, Dosage, DrugForm and Strength. Temporal attributes (e.g., frequency and duration) are expressed by means of temporal relations (not specific to drug entities). Frequency and dosage data are not split in atomic attributes for measurement units and values as can be seen on Fig. 1 (3rd sentence).
- Person attributes: we used a modified version of the *subject* attributes in SHARP (patient, family member, donor family member, donor other, and other) and the *subject class* templates in ShARe. We added the HealthProfessional value and simplified donor types. Some examples can be seen on Fig. 1 (1st sentence). We have also flagged coreferent pronouns, but only those referring to Person entities (e.g. *je*, ‘I’, refering to a physician). Coreference is annotated as a relation in CLEF, whereas coreference pronouns may refer to different entity types in i2b2, CLEF and SHARP. Lexical coreference is also annotated in i2b2, CLEF and SHARP, but not in our scheme. Note that coreference and anaphora are not annotated in MiPACQ. The annotation format in our scheme makes it possible to remove these flags easily and include or exclude them as a feature according to the training needs of a specific machine learning system.
- Measurements: Qualitative or quantitative descriptions of entities (adverbs, relational and qualitative adjectives, and quantifiers). We also consider measurement units for results of clinical tests, items in the *result* category from the Clinical E-Science Framework, and elements from the *lab result* semantic type within the *phenomena* class in the MiPACQ framework. The *severity* category (e.g. *grave*) lacks a specific label in our scheme, whereas the ShARe/CLEF eHealth labs and the SHARP Template Annotations encode it as an attribute. However, the measurement attribute in our scheme encompasses severity descriptors of entities together with other descriptions.
- Localization: This category expresses spatial details about entities (e.g. *droite*, ‘right’, or *inférieur*, ‘inferior’), which are often mapped to the UMLS Spatial concept type. This class subsumes attributes of SHARP (*body side, dorsal or ventral, medial or lateral, superior or inferior, distal or proximal*), and the *sub-location* and *laterality* modifiers from the Clinical E-Science Framework.

### Relations

**Fig. 4:**
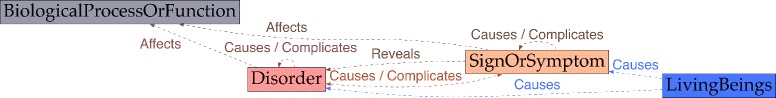
Relations starting from Disorder, SignOrSymptom and LivingBeing entities

**Fig. 5:**
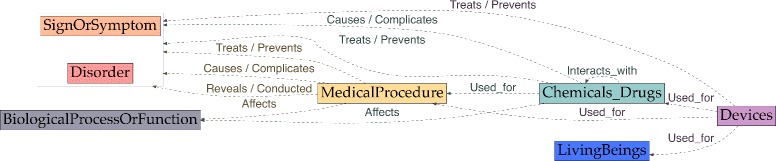
Relations starting from MedicalProcedure, Chemical_Drugs and Device entities

**Fig. 6:**
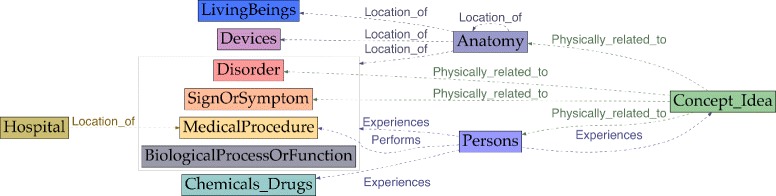
Relations starting from Anatomy, Persons, Concept_Idea and Hospital entities

Our scheme has 37 types of relations (Tables 4 and 5, Figs. 4, 5 and 6):

**Table 4:** Event-related relations

Relation	Definition	Involved entities
Affects	Produces a direct effect on a process or function	Disorder → BiologicalProcess SignOrSymptom → BiologicalProcess MedicalProcedure → BiologicalProcess Chemicals_Drugs → BiologicalProcess
Causes	Brings about a condition or an effect. Implied here is that an agent, such as a pharmacologic substance or an organism, has brought about the effect. This includes induces, effects, evokes and etiology	LivingBeings → Disorder LivingBeings → SignOrSymptom Chemicals_Drugs → Disorder Chemicals_Drugs → SignOrSymptom MedicalProcedure → Disorder MedicalProcedure → SignOrSymptom Disorder → Disorder SignOrSymptom → SignOrSymptom SignOrSymptom ⇔ Disorder
Complicates	Causes to become more severe or complex or results in adverse effects	Disorder → Disorder Chemicals_Drugs → Disorder MedicalProcedure → Disorder SignOrSymptom → SignOrSymptom Chemicals_Drugs → SignOrSymptom MedicalProcedure → SignOrSymptom SignOrSymptom ⇔ Disorder
Conducted	When a test is conducted to investigate a disorder and the outcome is unknown	MedicalProcedure → Disorder MedicalProcedure → SignOrSymptom
Experiences	When a human is affected by an event (e.g. a disorder or a medical procedure).	Persons → Disorder Persons → SignOrSymptom Persons → MedicalProcedure Persons → Chemicals_Drugs Persons → BiologicalProcess Persons → Concept_Idea
Interacts_with	Acts, functions, or operates together with	Chemicals_Drugs → Chemicals_Drugs
Localization_of	The spatial or relative localization of an entity	Localization → Entity
Location_of	The position, site, or region of an entity or the site of a process	Anatomy → Anatomy Anatomy → Disorder Anatomy → SignOrSymptom Anatomy → MedicalProcedure Anatomy → LivingBeings Hospital → MedicalProcedure
Measure_of	The relation between a measurement value and an entity	Measurement → event entity
Performs	A person conducts a procedure	Persons → MedicalProcedure
Physically_ Related_to	Related by virtue of some physical attribute or characteristic	Concept_Idea → Anatomy Concept_Idea → Persons Concept_Idea → Disorder Concept_Idea → SignOrSymptom
Prevents	Stops, hinders or eliminates an action or condition	Chemicals_Drugs → Disorder Chemicals_Drugs → SignOrSymptom MedicalProcedure → Disorder MedicalProcedure → SignOrSymptom Devices → Disorder Devices → SignOrSymptom
Reveals	When a test is conducted and the outcome is known or leads to a diagnosis	MedicalProcedure → Disorder MedicalProcedure → SignOrSymptom SignOrSymptom → Disorder
Treats	Applies a remedy with the object of effecting a cure or managing a condition	Chemicals_Drugs → Disorder Chemicals_Drugs → SignOrSymptom MedicalProcedure → Disorder MedicalProcedure → SignOrSymptom Devices → Disorder Devices → SignOrSymptom
Used_for	When a device is used (e.g. to conduct a treatment or to administer a drug)	Devices → MedicalProcedure Devices → Chemicals_Drugs Devices → LivingBeings

**Table 5:** Aspect, assertion, drug-attribute and temporal relations

Aspect	Definition	Involved entities
Continue	Shows the continuation of an event	Aspect → event entities
Decrease	A lowering value (e.g. of dose)	
Improve	An improvement (e.g. in condition)	
Increase	A rising value (e.g. of dose)	
Recurrence_ StartAgain	Indicates that an event begins occurring again	
Start	Indicates the initiation of an event	
Stop	Indicates the ending of an event	
Worsen	A negative change (e.g. in health)	
Assertion	Definition	Involved entities
Negation	An event is negated.	Assertion → event entities
Possible	An event may occur.	
Presence	An event occurs.	
SubjectToCondi- tion	An event may occur on condition that another event occurs	
Drug-attribute	Types	Involved entities
	HasAdministrationRoute	
	HasDosage	Chemical_Drugs → drug attributes
	HasDrugForm	
	HasStrength	
Temporal	Definition	Involved entities
Before	An event precedes another event/temporal expression	
Begins_on	The event starts on an event or temporal expression	Event entity → Event/Temporal entity
During	The temporal span of an event is completely contained within the span of another event or temporal expression	
Ends_on	The event finishes on an event or temporal expression	
Overlap	An event happens almost at the same time, but not exactly, as another event/temporal expression	
Simultaneous	An event happens at exactly the same time as another event/temporal expression

- Aspect relations: they encode a change (or lack of change) with regard to an entity: Continue, Decrease, Improve, Increase, Recurrence_StartAgain, Start, Stop and Worsen. They were inspired by *aspectual* events in the THYME scheme, and *status change* or *course class* attributes in SHARP. For instance, in the phrase *introduction de l’EPO le 12/04/2012* (‘EPO was introduced on 04/12/2012’), the mention *introduction* (‘introduced’) characterizes a status change with respect to the EPO therapy, as explained above. The relation Start further qualifies the change experienced. This is an information different in nature compared to the temporal information found in the second part of the phrase *le 12/04/2012* (‘on 04/12/2012’), which anchors the therapy event on a timeline. Therefore, the second part of the phrase is annotated with a Temporal entity with the modality Date (*12/04/2012*) and a Begin_on relation can be used to represent the temporal link between the Chemicals_Drugs event *EPO* and the Date *12/04/2012*.
- Assertion relations: Negation, Possible, Presence and SubjectToCondition. These are a subset of the i2b2 assertion challenge, but we removed the *Not associated with the patient* i2b2 assertion. The notion that an event is not associated with the patient is conveyed by its being associated with another Person (e.g. HealthProfessional, ‘PatientFamily’) through the relation Experiences. An example of an assertion relation can be found in the phrase *pas de douleur* (‘no pain’), where the mention *pas de* (‘no’) can be represented as a textual marker linked to the SignOrSymptom concept *douleur* (‘pain’) using a Negation relation. The only assertion that may not have an explicit textual marker to anchor the relation is Presence. When a textual marker is found, the relation can be represented: e.g. *présence de* (‘presence of’) in the phrase *présence de marisques* (‘presence of hemorrhoidal tags’). When there is no textual marker, no relation can be represented—however, it is likely that entities without an explicit assertion are implicitly asserted as Presence.
  Unlike the i2b2 challenge, assertions can be made on any entity (as in [23]).
- Drug-attribute relations: four types of links to medication attributes: HasAdministrationRoute, HasDosage, HasDrugForm and HasStrengh.
- Temporal relations: six types of chronological relationships: Before, Begins_on, During, Ends_on, Overlap and Simultaneous (Fig. 3).
- Event-related relations: there are 15 types (Table 4).

### Time representation

The temporal scheme for annotation was derived from TimeML [44], and comprises four types of Temporal Expressions (encoded with the attribute TemporalType): Date, Time, Frequency and Duration. Event entities may be related to other events or to temporal expressions by means of the temporal relations described above. Finally, temporal data is also encoded for each event entity by means of the DocTime attributes.

Figure 3 represents the temporal elements in our scheme.

### Sample application

We applied the annotation scheme described herein to the development of a large French clinical corpus as detailed in [13]. The BRAT [60] configuration files for the annotation schemes are available for the research community. Temporal representation in this corpus differs slightly from TimeML in that signals were annotated together with temporal expressions instead of being annotated separately. For instance, the entire expression *il y a 5 ans* (‘five years ago’) was annotated as a time expression of the type duration, while strict TimeML guidelines would require *5 ans* (‘5 years’) to be annotated as a Duration and *il y a* (‘ago’) to be annotated as a signal. This minor divergence was initially implemented as a shortcut to limit the burden of carrying out complex annotations in the course of an annotation project relying on the complete scheme. However, later work that focused on time analysis could easily revert to standard TimeML and show a compatibility between the representations [61, 62].

## Discussion

### Characteristics of existing information schemes

**Fig. 7:**
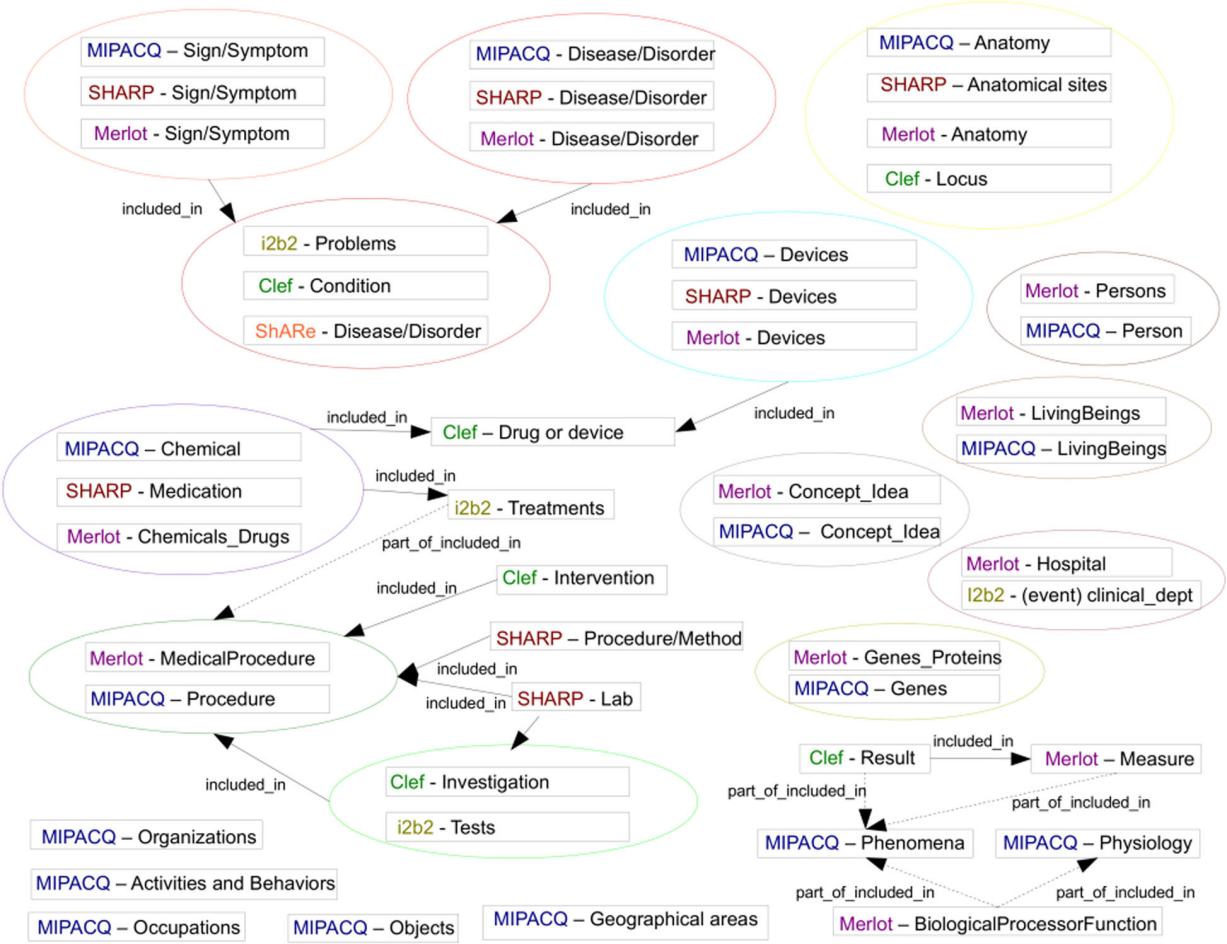
Entities in existing annotation schemes

**Fig. 8:**
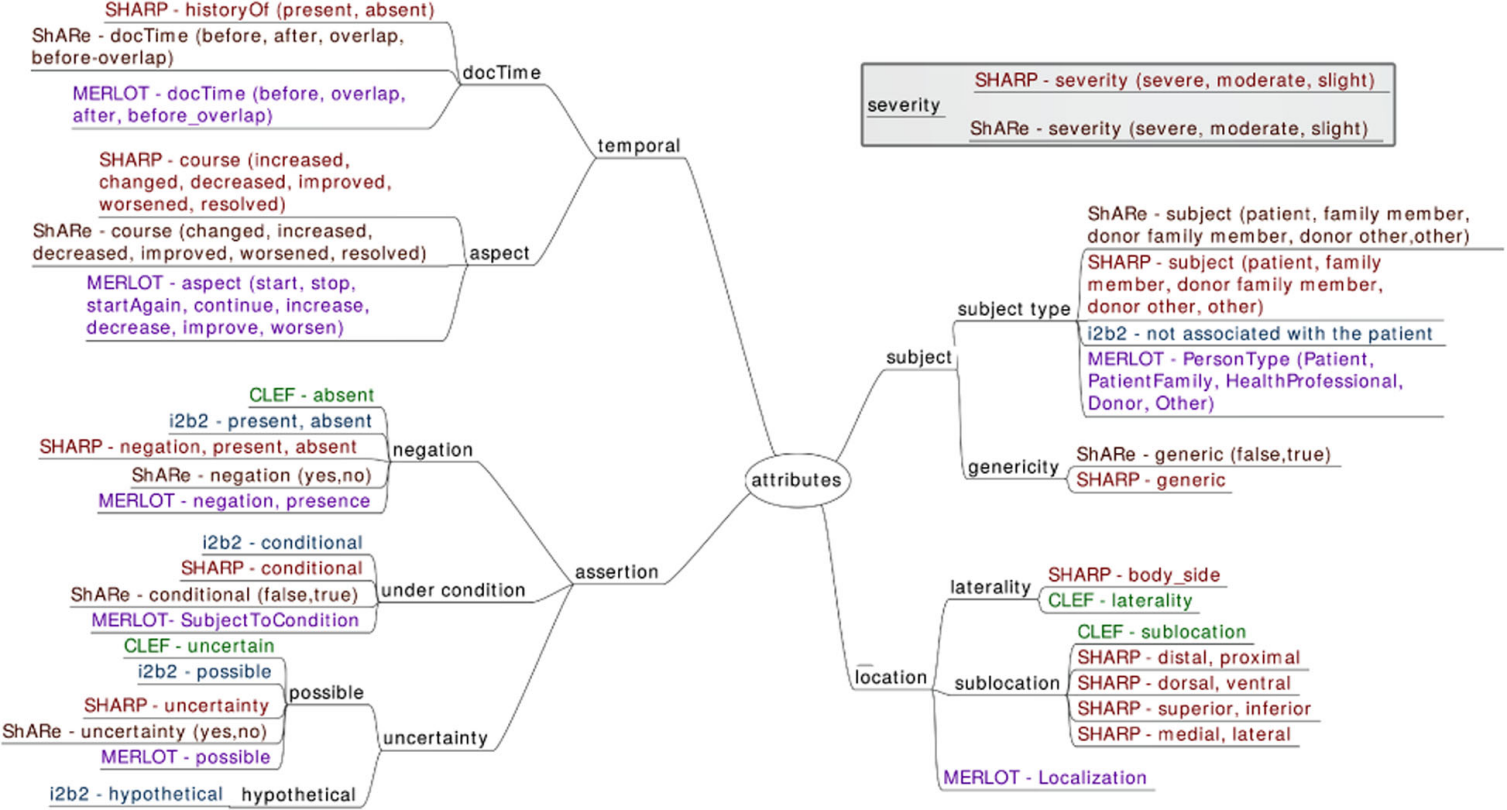
Attributes in existing annotation schemes

**Fig. 9:**
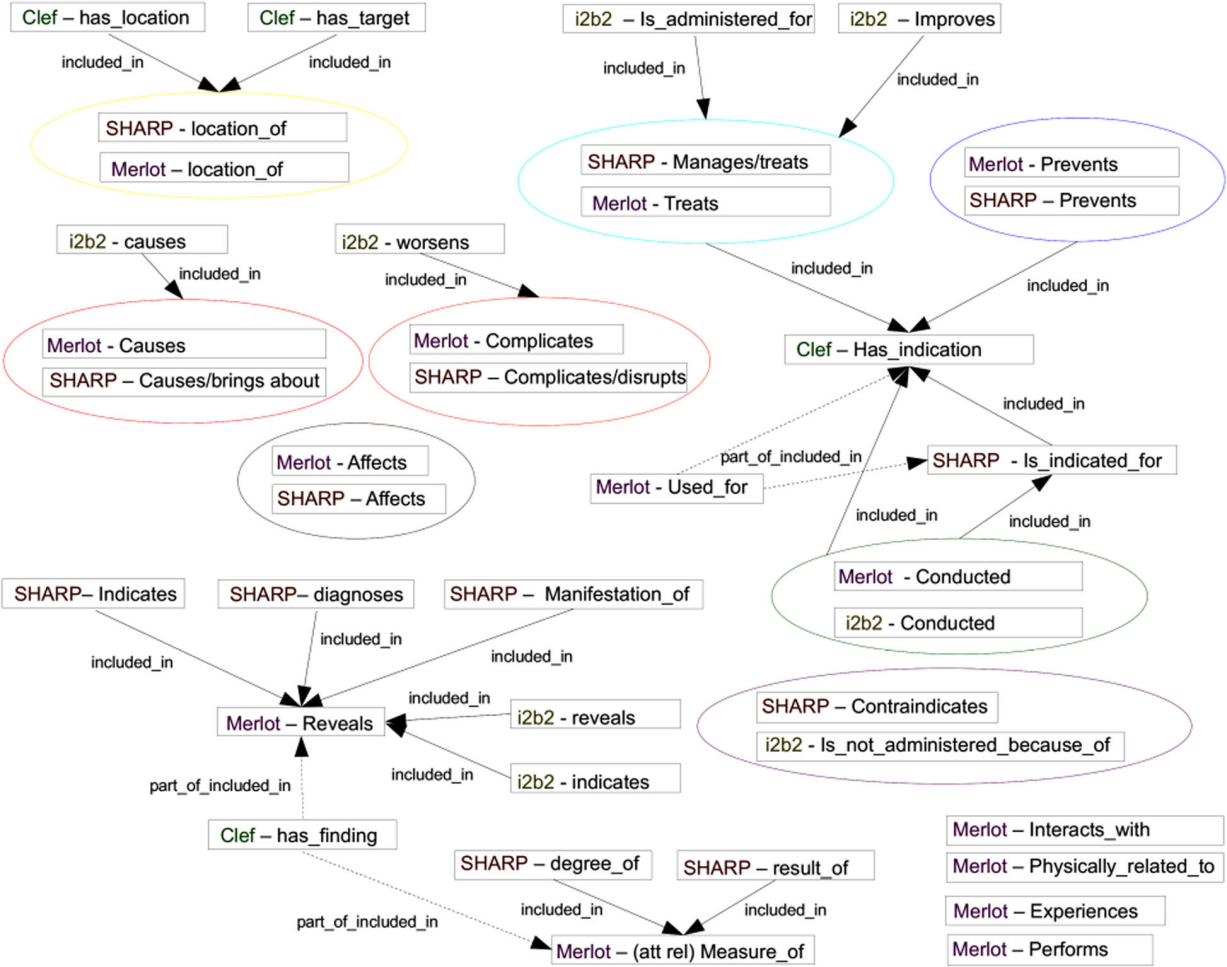
Relations in existing annotation schemes

One important criterion to consider in the choice of an information scheme is undoubtedly whether it covers the immediate information needs of the project. Depending on the expected growth of the work-flow, the evolution or life cycle of the scheme should also be taken into account. Our analysis of existing annotation schemes provides an overview of the entities, attributes and relations covered in the literature. It can be seen that MIPACQ is one of the most extensive with regard to entities (Fig. 7); ShARe and SHARP provide a very refined set of attributes (Fig. 8); and our scheme offers a large set of relations, including temporal relations (Fig. 9 and Tables 4 and 5). Nevertheless, an essential in ontologies or information schemes is whether they have been designed as application-dependent, application-semidependent or application-independent [63]. As we illustrate below, our information scheme was built with an application-semidependent perspective.

### Quality of the information representation

Our scheme is rather robust to cope with gastro-enterology as well as other specialities, including foetopathology or nephrology, as tested in preliminary work [57, 61]. The overall inter-annotator agreement observed over 11 annotator pairs is 0.793 for entities and 0.789 for relations in a recent annotation campaign using this annotation scheme [13]. The soundness of our scheme was further assessed by the review of a medical doctor, who applied the scheme to a small set of training documents. He achieved an F-measure of 0.720 with regard to the consensus annotations of entities and 0.740 for relations, which was in the range of F-measure values for the rest of annotators. Overall, the inter-annotator agreements obtained using the scheme are good, demonstrating that the information representation encoded is robust and can be applied consistently.

### Use cases and information extraction scenarios

Our goal is to put forth a representation scheme that will support secondary use of EHR data for conducting a large variety of retrospective studies. More specifically, we aim to support information extraction from clinical narratives in order to answer clinical questions such as: “What is the prevalence of incidental findings in patients with suspected thromboembolic disease?”, or “What are the types and grades of toxicities experienced by colon cancer patients receiving FOLFOX therapy?”. The first type of question fits the document-level classification scenario. That is to say, relevant documents within patient records will need to be classified as consistent with incidental finding and thromboembolic disease suspicion. The aggregation of results over a large number of records is then expected to address the prevalence. The second type of question fits the information extraction scenario where the toxicities and their characteristics need to be retrieved from relevant text passages within the records. We bear in mind, nonetheless, that the scope of the results will always be biased by the corpus data and its representativeness.

Other teams have already made valuable contributions after using annotation schemes to describe the information within the clinical narrative [64]. All have reported positive results, especially with regard to increasing annotation agreement, recognizing document sections where specific information is communicated, and improving clinical information retrieval.

An example is the scheme devised by [26], which encodes both syntactic and semantic (domain knowledge) descriptions of clinical conditions (linguistic form, modifier types and medical concepts). They applied it to improve the indexing of clinical conditions in emergency department reports, and observed that using the annotation scheme significantly increased agreement in annotations as compared to a baseline scheme [27].

For its part, the scheme developed by [28] comprised entities and attributes to encode phenotypic information of inflammatory bowel disease. With the help of the scheme, this team identified the types of information structure and section types in EHRs where the highest number of concepts were expressed.

Finally, several teams have made positive contributions when transposing an annotation scheme to a knowledge representation for clinical information retrieval. Some schemes have been applied for creating gold standard corpora to develop and evaluate clinical information extraction tools [15] and entity recognition systems [17, 18]. These reference corpora have also been used for relation extraction; an example is the work on mining adverse reactions by means of machine learning reported by [19]. The scheme devised by [11] was used to both define the features for statistical classification models—which improved performance over bag-of-word approaches—and describe implicit data within the clinical narrative. For example, the diagnosis method was not explicitly written in radiology reports, but rather had to be inferred from the type of exam.

### Limitations of the representation scheme

A limitation in our scheme is the fact that it does not fully integrate the same level of granularity for annotating certain entities or attributes as other frameworks do.

An example is the *localization* category in our scheme, which is a broad class subsuming the *sub-location* and *laterality* attributes in the Clinical E-Science Framework and lacks the precision of the SHARP attributes. These attributes modify entities in the *anatomy* category (called *Locus* in CLEF). The SHARP annotation scheme is disease-centric, so that anatomical information is considered as an attribute—called *body location*—of an annotated disease or condition. The SHARP guidelines encode further descriptive attributes according to spatial axes: *body side* (left, right, bilateral, unmarked), *distal or proximal* (distal, proximal, unmarked), *dorsal or ventral* (dorsal, ventral, unmarked), *medial or lateral* (medial, lateral, unmarked), and *superior or inferior* (superior, inferior, unmarked). That is, our scheme, CLEF and SHARP encode these data, but CLEF and SHARP allow the automatic extraction of localization details in a more specific and direct way.

Another example is the *severity* attribute in the ShARe and SHARP guidelines, which lacks a label in our scheme, but is subsumed in a broader category (Measurement). Refining these categories is important to specify and describe more relation types. This would permit our scheme to provide a better representation of information across clinical areas and text genres in more robust manner. For example, radiology reports especially require fine-grained categorizations of location relationships. These shortcomings need to be addressed and explored in the future.

Our annotation scheme could also be improved further to encode coreference and anaphoric relations in a more precise manner. We could build on annotation frameworks already existing in the clinical [65] and the biological domain [66].

Despite the aforementioned weaknesses, we would like to highlight the robustness and coherence of our annotation framework. In future endeavours, this will allow us to integrate our scheme into computer-readable encoding standards (e.g. XML), as current research teams have carried out [67]. We devised the scheme in a *traditional annotation framework* [30]—i.e. guidelines were developed and refined through training and double annotation, and after consecutive rounds of annotations and consensus. However, the conceptual model could be used in other annotation frameworks based on crowd-sourced annotation [68] and community annotation [45].

## Conclusion

We have described the development of a broad-scope annotation scheme intended to support information extraction from Electronic Health Records for retrospective studies and translational research. Our comparative review of the literature shows that the scheme covers most of the information also described in other frameworks, and especially features a rich range of relations. The scheme and companion material (annotation scheme and configuration files) are freely available to the community.
